# Myths and methodologies: Cardiopulmonary exercise testing for surgical risk stratification in patients with an abdominal aortic aneurysm; balancing risk over benefit

**DOI:** 10.1113/EP090816

**Published:** 2023-05-26

**Authors:** Damian M. Bailey, Richard G. Davies, George A. Rose, Michael H. Lewis, Ahmed Abd Aldayem, Chistopher P. Twine, Wael Awad, Matti Jubouri, Idhrees Mohammed, Carlos A. Mestres, Edward P. Chen, Joseph S. Coselli, Ian M. Williams, Mohamad Bashir, The International Academic Surgical Consortium (THALAMUS)

**Affiliations:** ^1^ Neurovascular Research Laboratory, Faculty of Life Sciences and Education University of South Wales Glamorgan UK; ^2^ Department of Anaesthetics University Hospital of Wales Cardiff UK; ^3^ Department of Vascular Surgery University Hospital of Wales Cardiff UK; ^4^ Department of Surgery University of Bristol Bristol UK; ^5^ Department of Cardiothoracic Surgery Bart's Heart Centre, St Bartholomew's Hospital, Bart's Health NHS Trust London UK; ^6^ Hull York Medical School University of York York UK; ^7^ Institute of Cardiac and Aortic Disorders SRM Institutes for Medical Science (SIMS Hospital) Chennai Tamil Nadu India; ^8^ Department of Cardiac Surgery University Hospital Zürich Zürich Switzerland; ^9^ Division of Cardiovascular and Thoracic Surgery Duke University Medical Center Durham North Carolina USA; ^10^ Division of Cardiothoracic Surgery, Michael E. DeBakey Department of Surgery Baylor College of Medicine Houston Texas USA; ^11^ The Texas Heart Institute Houston Texas USA; ^12^ St Luke's‐Baylor St. Luke's Medical Center Houston Texas USA; ^13^ Vascular and Endovascular Surgery Health & Education Improvement Wales Cardiff UK

**Keywords:** abdominal aortic aneurysm, cardiopulmonary exercise testing, perioperative outcome, rupture, surgical risk stratification

## Abstract

The extent to which patients with an abdominal aortic aneurysm (AAA) should exercise remains unclear, given theoretical concerns over the perceived risk of blood pressure‐induced rupture, which is often catastrophic. This is especially pertinent during cardiopulmonary exercise testing, when patients are required to perform incremental exercise to symptom‐limited exhaustion for the determination of cardiorespiratory fitness. This multimodal metric is being used increasingly as a complementary diagnostic tool to inform risk stratification and subsequent management of patients undergoing AAA surgery. In this review, we bring together a multidisciplinary group of physiologists, exercise scientists, anaesthetists, radiologists and surgeons to challenge the enduring ‘myth’ that AAA patients should be fearful of and avoid rigorous exercise. On the contrary, by appraising fundamental vascular mechanobiological forces associated with exercise, in conjunction with ‘methodological’ recommendations for risk mitigation specific to this patient population, we highlight that the benefits conferred by cardiopulmonary exercise testing and exercise training across the continuum of intensity far outweigh the short‐term risks posed by potential AAA rupture.

There are in fact two things, science and opinion; the former begets knowledge, the latter, ignorance.

Hippocrates, 400 CE

## 1 CRUX OF THE CONUNDRUM: EXERCISING PATIENTS WITH AN ABDOMINAL AORTIC ANEURYSM

There is overwhelming clinical and scientific evidence to indicate that exercise training is as good as, if not indeed more effective than, any other medicine used to treat chronic diseases affecting the cardiopulmonary, cerebral and metabolic circulation; simply put, those who are prescribed it live longer and enjoy a better quality of life (Franklin et al., 2020), with any increase in physical activity positively impacting cardiorespiratory fitness (CRF) and reducing mortality risk (Grandes et al., 2023). However, its prescription, like any other medicine encompassing complex interactions between dosage and formulation, needs to take into account the clinical status of the patient, including pre‐existing cardiovascular, pulmonary, cerebrovascular and metabolic diseases that have implications for exercise risk stratification/mitigation (Thompson et al., 2020). Despite its long‐term cardiovascular, pulmonary, cerebrovascular and metabolic benefits, acute vigorous and (ultra)long‐duration moderate‐intensity exercise, especially when performed by those who are unfit, has the potential to cause harm, as demonstrated by the increased incidence of sudden cardiac death and acute myocardial infarction (MI) (Pelliccia et al., 2021); the paradox of the panacea!

More recently, concerns have been extended to patients diagnosed with an abdominal aortic aneurysm (AAA), inspired more by intuitive speculation than empirical evidence, owing to the perceived increased risk of blood pressure (BP)‐induced expansion leading to dissection or rupture, which typically proves catastrophic (Myers et al., 2012). This is especially relevant when AAA patients undergo cardiopulmonary exercise testing (CPET), which, by design, involves incremental exercise to symptom‐limited exhaustion for determination of CRF, a rapidly evolving ‘vital sign’ (Ross et al., 2016) that can inform perioperative risk (Rose et al., 2022).

In this ‘Myths and Methodologies’ review, we bring together a multidisciplinary group of physiologists, exercise scientists, anaesthetists, radiologists and surgeons to challenge the enduring ‘myth’ that AAA patients should be fearful of and avoid rigorous exercise. On the contrary, by appraising fundamental vascular mechanobiological forces associated with exercise together with newly formulated clinical recommendations for risk mitigation, we highlight that the benefits conferred by CPET and exercise training across the continuum of intensity far outweigh the short‐term risks posed by potential rupture.

## 2 ABDOMINAL AORTIC ANEURYSM: PRESENTATION, DIAGNOSIS, RISKS AND REPAIR

**FIGURE 1:**
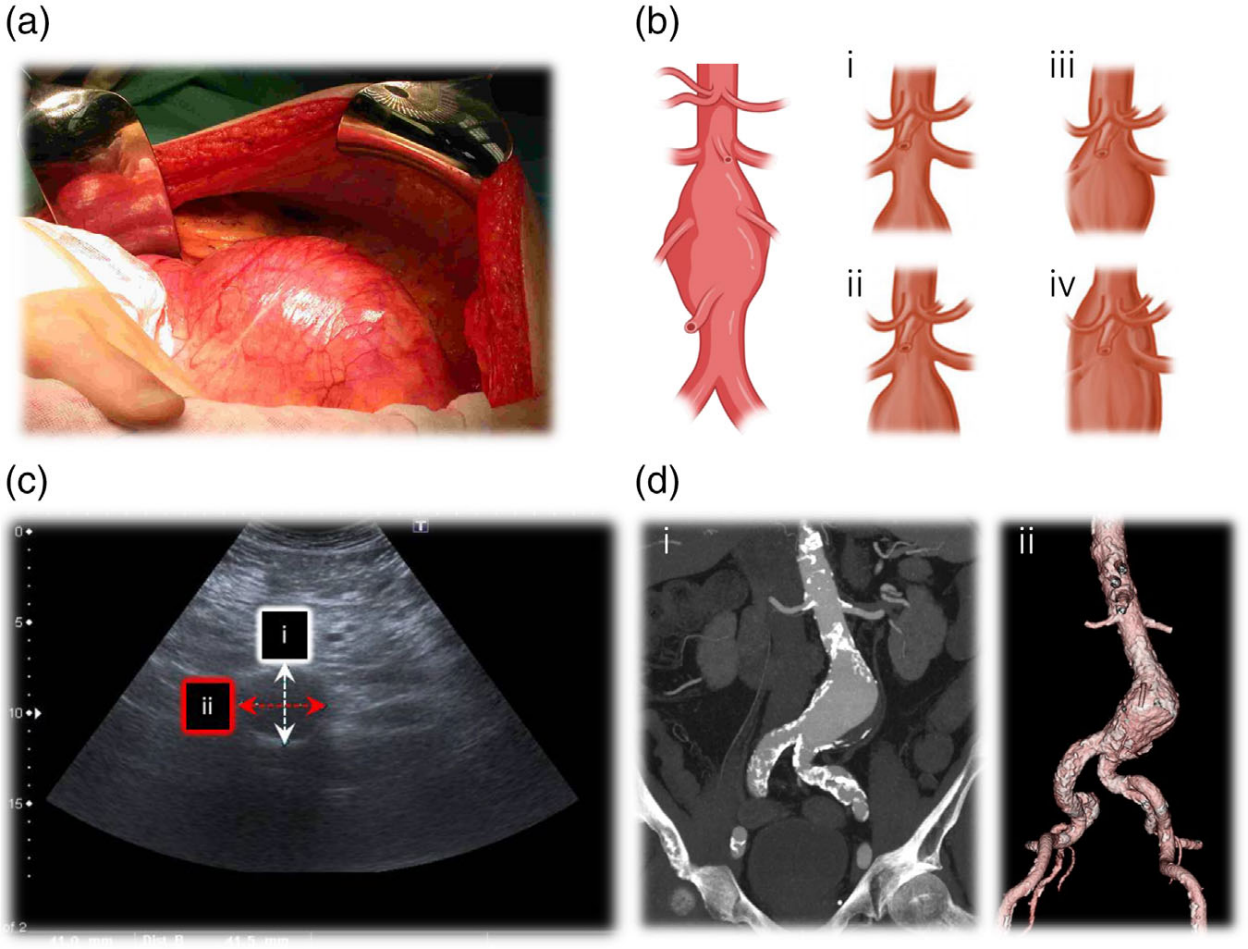
Clinical presentation of an abdominal aortic aneurysm (AAA). (a) Surgical appearance of a large (8.5‐cm‐diameter) infrarenal AAA during open transperitoneal repair. (b) Anatomical classification of AAAs: i, infrarenal AAA; ii, juxtarenal AAA; iii, suprarenal AAA; iv, type IV thoracoabdominal aortic aneurysm. (c) Appearance on ultrasound (transverse plane), enabling assessment of anteroposterior (i) and transverse (ii) diameters. (d) Coronal maximum‐intensity projection (i) and three‐dimensional reformat (ii) of an AAA (5.5 cm in diameter). Note the adequate length of the iliac arteries and the long infrarenal neck below the renal arteries, highlighting suitability for endovascular repair. (d) Adapted from (Swerdlow et al., 2019).

The word ‘aneurysm’ is derived from the Greek word ‘aneurysma’, meaning ‘dilatation’, and in the setting of AAA it reflects the abnormal geometric expansion of the abdominal aorta to 1.5 times its expected (anteroposterior) diameter, >3.0 cm (Figure 1a). Abdominal aortic aneurysms (AAAs) are classified anatomically as infrarenal, juxtarenal, suprarenal or thoracic, with ∼90% originating below the renal arteries (Figure 1b), as the consequence of a specific flow phenotype (see Section 5 PHYSIOLOGY OF FLOW AND RISK OF RUPTURE: YIN AND YANG). Ultrasound imaging remains the first‐line investigation for initial diagnosis and surveillance screening (Figure 1c), with computed tomography (CT), angiography or magnetic resonance imaging to complement operative planning (Figure 1d).

**FIGURE 2:**
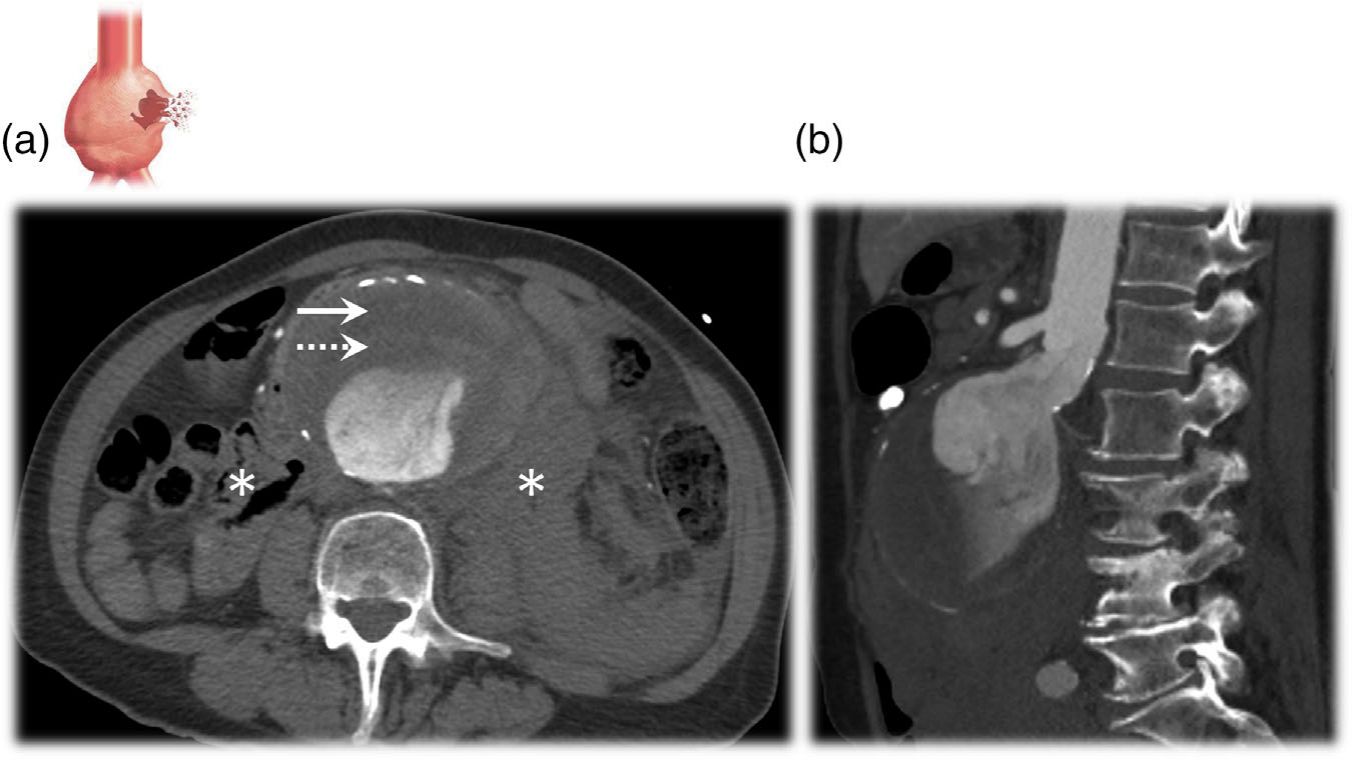
Contrast‐enhanced computed tomography scans of a ruptured abdominal aortic aneurysm (AAA). The scans illustrate a large (9.0‐cm‐diameter) ruptured AAA in a single patient. (a) Axial plane; note the crescent sign (rim of hyperdensity, continuous arrow) within the mural thrombus (dotted arrow), with a retroperitoneal haematoma visible anterior to the psoas muscles (asterisks). (b) Sagittal plane.

**FIGURE 3:**
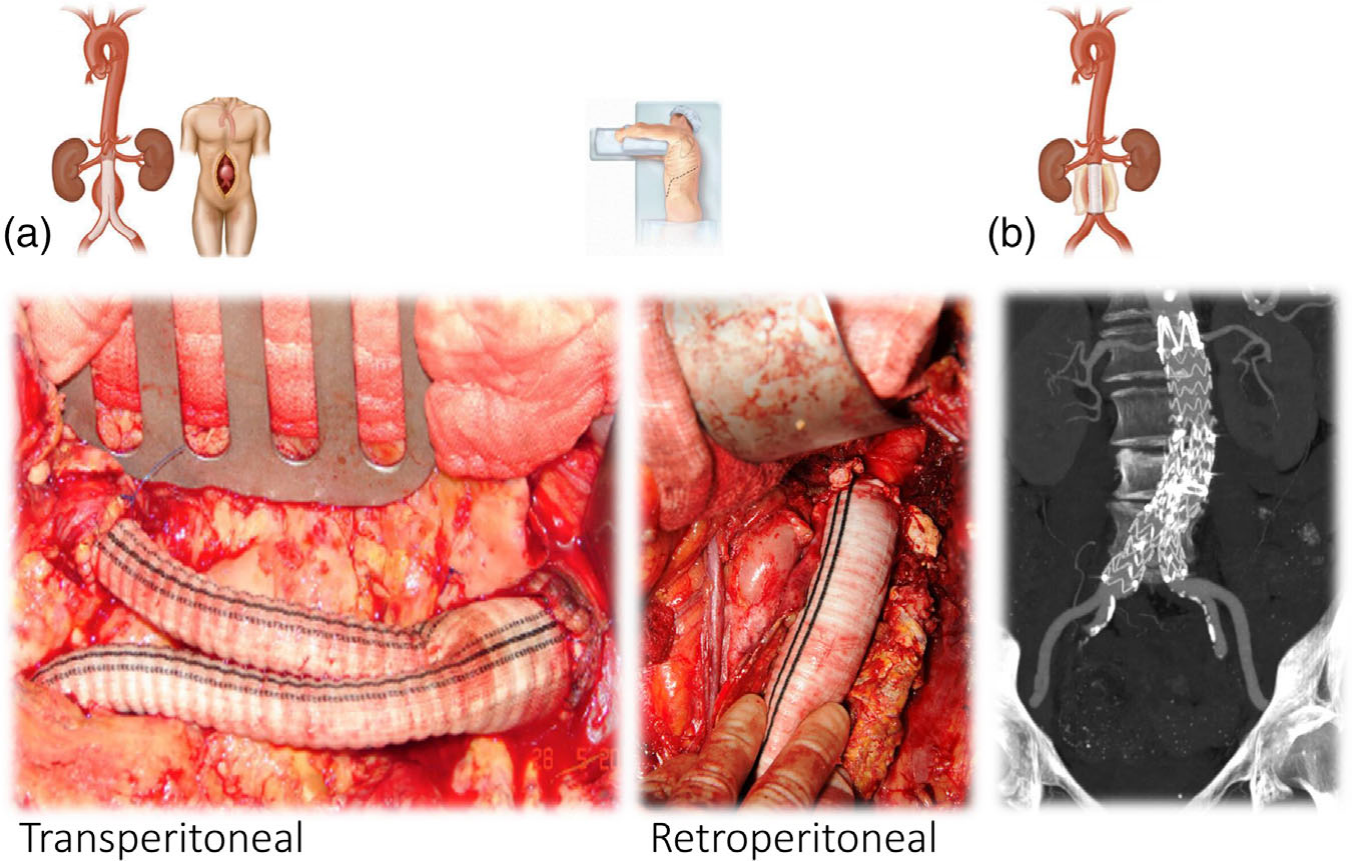
Open repair and endovascular repair of abdominal aortic aneurysm (AAA). (a) Left panel, open transperitoneal AAA repair, showing a bifurcated synthetic prosthetic graft, with a proximal anastomosis at the infrarenal neck and distally to the origin of both common iliac arteries. (a) Right panel, open retroperitoneal repair of a juxtarenal AAA with the duodenum and left ureter seen to the left of the open aortic sac. (b) Coronal maximum‐intensity projection, with endovascular stent graft deployed. Note uncovered struts above the renal arteries and covered extensions into both iliac arteries to ensure that no endoleak develops.

The most serious complication associated with AAAs is rupture (Figure 2a,b), with mortality estimated between 50% and 90% (Kent, 2014), accounting for 167,200 deaths and 3 million disability‐adjusted life years in 2017 (GBD 2017 Causes of Death Collaborators). However, the risk of rupture for patients under surveillance with small (3.0−4.4 cm) or medium‐sized (4.5−5.4 cm) AAAs is low, estimated in the order of <0.5% per annum (Oliver‐Williams et al., 2019). Elective surgery is the preferred treatment option to reduce the future risk of rupture in patients with large (male ≥ 5.5 cm, female ≥ 5.0 cm), rapidly growing (1.0 cm year^−1^) or symptomatic AAAs. This involves either open surgical repair, whereby the aneurysmal sac is replaced with a vascular synthetic graft (Figure 3a), or endovascular repair, which involves excluding the AAA from the systemic circulation with a stent graft (Figure 3b) (NICE, 2020). However, surgery is not without risk, with in‐hospital mortalities of 3.1% and 0.5% (infrarenal AAAs) and 10.9% and 2.6% (suprarenal AAAs) reported for open surgical repair and endovascular repair, respectively, in the UK between 2019 and 2021 (Waton et al., 2022).

## 3 CARDIOPULMONARY EXERCISE TESTING: SURGICAL ‘SURVIVAL OF THE FITTEST’

Cardiopulmonary exercise testing is internationally recognized as the gold standard for the objective evaluation of functional capacity and CRF. Typically, it involves a maximal exercise stress test with concomitant breath‐by‐breath respiratory gas exchange analysis for the assessment of specific metrics including, albeit not exclusively confined to the following: peak oxygen uptake (${\dot{V}}_{O_{2}\mathrm{peak}}$), gas exchange threshold (GET), ventilatory equivalent for carbon dioxide output (${\dot{V}}_{E}/{\dot{V}}_{CO_{2}}$), O_2_ pulse, O_2_ uptake efficiency slope, combined with integrated evaluation of heart rate (HR), including the chronotropic response to and recovery from progressive exercise, blood pressure (BP), oxygen saturation and (12‐lead) ECG. The practical assessment of these multimodal metrics detailing indications, organization, conduct and physiological interpretation, including relative sensitivity for risk prediction as a function of disease state (including chronic obstructive pulmonary disorder, chronic heart failure, interstitial lung disease, cystic fibrosis and pulmonary hypertension) and (planned) surgical intervention, have been reviewed extensively (Laveneziana et al., 2021; Levett et al., 2018; Older & Levett, 2017; Otto et al., 2020).

After seminal work in the early 1990s (Older et al., 1993), CPET has become widely adopted as a complementary clinical tool that can detect functional anomalies and long‐term sequelae of individualized comorbidities that are ‘unmasked’ by the increasing systemic O_2_ demands of progressive exercise, helping to identify those patients at increased risk of perioperative events during AAA surgery (Grant et al., 2015; Rose, Davies, Appadurai et al., 2018, Rose, Davies, Davison et al., 2018). Increased basal O_2_ demand is commonly observed during the intra‐ and postoperative phases of AAA repair (Ciaffoni et al., 2016), with basal metabolic rate elevated as high as three times preoperative values (Older & Smith, 1988; Viale et al., 1991), which might be related to a systemic elevation in oxidative–inflammatory–nitrosative stress (OXINOS), defined by a free radical‐mediated amplification of inflammation and concomitant reduction in vascular nitric oxide (NO) bioavailability, endothelial dysfunction and compromised tissue integrity (Bailey et al., 2006, 2022).

Thus, unlike static measurements historically constrained to single organ function, the multimodal assessment of CRF better reflects the integrated physiological capacity of a patient or their ‘resilience’ to meet the physiological homeostatic challenge associated with this increased O_2_ demand that ultimately allows the AAA patient to survive surgical repair. In support, numerous studies have since identified that select metrics (${\dot{V}}_{O_{2}\mathrm{peak}}$ ≤ 15.0 mL kg^−1^ min^−1^ and GET ≤ 10.2 mL kg^−1^ min^−1^) are significant predictors of short‐ and long‐term postoperative mortality/morbidity in AAA patients regardless of repair modality (Perissiou et al., 2022). However, preoperative risk stratification can be improved further if a combination of ${\dot{V}}_{O_{2}\mathrm{peak}}$, GET and ${\dot{V}}_{E}/{\dot{V}}_{CO_{2}}$ are used (Grant et al., 2015). However, it remains to be established to what extent other metrics, including combinations thereof and corresponding ‘cut‐offs’ or threshold boundaries, collectively serve to optimize risk stratification further across the perioperative continuum in AAA patients.

Regardless, it is now estimated that >30,000 CPET tests are conducted annually in the UK alone (Reeves et al., 2018), with ∼83% of all AAA patients undergoing CPET as part of their integrated preoperative risk assessment (Waton et al., 2022). Clinicians have become increasingly reliant on CRF metrics to inform and guide the decision to proceed to surgery, triage to the appropriate level of care, optimize anaesthetic techniques, diagnose unexpected comorbidities and, increasingly, to tailor individualized preoperative exercise programmes as part of prehabilitation (Perissiou et al., 2022; Rose et al., 2022).

## 4 CHALLENGING THE EXERCISE MYTH: RUNNING (AGAINST) THE RISK OF ABDOMINAL AORTIC ANEURYSM RUPTURE

Informed more by theoretical concerns than empirical evidence, the American College of Sports Medicine (ACSM) guidelines originally recommended that AAA patients should not undergo maximal exercise testing and that HR should be maintained <100 beats min^−1^, to avoid excessive increases in the rate–pressure product (not routinely used for CPET termination), which can increase the potential risk of AAA expansion and rupture (Pyeritz, 1997). Hence, preoperative risk assessments have traditionally relied on pharmacological stress testing (e.g., dobutamine) as a more controlled, safer intervention.

Given that CPET typically requires a maximal volitional effort for ‘optimized’ risk prediction (see Section 6 CLINICAL RECOMMENDATIONS: INTEGRATED PHYSIOLOGY‐INFORMED BEST PRACTICE), it is associated with an increased risk of inducing syncope, hypoxaemia or malignant cardiac dysrhythmias and of exacerbating previously latent conditions, especially in patients with pre‐existing comorbidities (Pritchard et al., 2021). However, the evidence to date suggests that CPET is, generally, a safe procedure, assuming strict adherence to quality‐assured standards and safety recommendations, including absolute/relative contraindications to exercise that serve collectively to mitigate these risks significantly (Levett et al., 2018). In support, complication rates that require hospitalization are estimated at ≤2 in 1000, with major cardiac events of 1.2 per 10,000 tests and mortality of 2–5 per 100,000 tests, with no deaths reported to the best of our knowledge in the UK (Levett et al., 2018; Pritchard et al., 2021).

Long‐term moderate‐intensity continuous (exercise) training (MICT) interventions (typically ranging from 6 to 12 weeks, two or three exercise bouts per week) as part of prehabilitation are considered safe and well tolerated in AAA patients. In support, a recent meta‐analysis involving seven trials, with a combined total of 489 patients, documented a cardiovascular event rate of only 0.8% (Kato et al., 2019), which is (almost counterintuitively) half that reported in healthy older participants without AAA (1.5%) (Goodyear et al., 2013). Importantly, MICT has been shown to improve GET by 1.1−3.0 mL kg^−1^ min^−1^ (Barakat et al., 2016; Kothmann et al., 2009; Tew et al., 2012) and ${\dot{V}}_{O_{2}\mathrm{peak}}$ by 1.2−1.7 mL kg^−1^ min^−1^ (Barakat et al., 2016; Lima et al., 2018; Tew et al., 2012) compared to usual care controls.

However, despite these improvements in CRF, the evidence for benefit is not clear enough for exercise training to be recommended in clinical guidelines (Perissiou et al., 2022) and (Tew et al., 2022). High‐intensity interval exercise training (HIIT), characterized by intermittent bouts of severe (i.e., in excess of the GET) exertion lasting 2–4 min interspersed with equivalent recovery intervals of either low‐intensity exercise or complete rest, was recently shown to be safe in patients with large AAAs (≤7.0 cm), assuming strict adherence to safety guidelines (systolic BP < 180 mmHg and/or HR < 95% of the maximum observed on baseline CPET) (Weston et al., 2016). This might prove a more effective intervention, given that its characteristic sinusoidal flow–shear–strain phenotype can further potentiate molecular, metabolic, haemodynamic and structural adaptations to exercise (Calverley et al., 2020). In support, studies in healthy participants (mean ${\dot{V}}_{O_{2}\mathrm{peak}}$ ranging between 41 and 61 mL O_2_ kg^−1^ min^−1^) and patients with established cardiometabolic disease (mean ${\dot{V}}_{O_{2}\mathrm{peak}}$ of 23 mL O_2_ kg^−1^ min^−1^) have consistently demonstrated greater increases in ${\dot{V}}_{O_{2}\mathrm{peak}}$of the order of ∼1.7 mL O_2_ kg^−1^ min^−1^ after HIIT compared with an equivalent volume of MICT (Helgerud et al., 2007; Milanovic et al., 2015; Weston et al., 2014).

In contrast, comparatively fewer studies have addressed the safety aspects of maximal exercise testing (CPET) in AAA patients, owing, at least in part, to the fact that many of the AAAs are likely to have escaped formal diagnosis, especially in the pre‐screening era (2009 in the UK) (Myers et al., 2012). Equally, although indications, complications and contraindications to exercise in patients with cardiovascular disease are generally well defined, there is little, if any, specific reference in the literature on whether CPET is contraindicated or not in AAA patients, with some guidelines quoting arbitrary geometric cut‐points in the absence of scientific evidence (Levett et al., 2018). It is conceivable that patients with occult small AAAs (those not yet meeting the diameter‐based threshold of repair; e.g., 3.0–5.5 cm) have undoubtedly performed CPET and/or participated in exercise training programmes during cardiac rehabilitation.

Regardless, the evidence to date suggests that maximal exercise in these patients is equally safe and carries minimal risks, despite many centres continuing to opt for submaximal and/or pharmacological stress testing to risk stratify patients owing to theoretical safety concerns of rupture. In support, one of the largest studies conducted to date compared 306 AAA patients (AAAs ≥3.0 and ≤5.0 cm) with 2155 age‐matched veterans referred for an exercise stress test for other reasons (Myers et al., 2011). Mean ${\dot{V}}_{O_{2}\mathrm{peak}}$ and peak HR were comparable between groups, and athough the exercise‐induced elevation in systolic BP was more marked in AAA patients, the rate of increase was considered normal (8–10 mmHg MET^−1^). Furthermore, although AAA patients experienced a higher frequency of hypertension, no major adverse clinical events were reported.

To date, there have been three cases documented of AAA rupture during exercise stress testing (Puls & Thadani, 1986; Best et al., 1998; Robertson et al., 2017) and one reporting delayed (3 days after CPET) splenic rupture (Davies et al., 2016) in the literature, although a direct causal link with exercise remains controversial.

The earliest case report of AAA rupture was documented by Puls & Thadani (1986) in a 71‐year‐old male with an asymptomatic AAA 7.0 cm in diamter and history of hypertension/MI, who underwent nuclear ventriculography during supine cycle ergometry. Six minutes into the test, at a power output of ∼65 W, HR of 115 beats min^−1^ and BP of 200/100 mmHg, he developed acute lower abdominal and back pain. The test was discontinued immediately, and he became hypotensive, with subsequent ultrasonography confirming rupture, and the patient underwent emergency open surgical repair but died from MI 2 days later. The authors highlighted the need to monitor exercise BP and to be cautious when exercising patients with large AAAs (i.e., those above the operative threshold diameter).

Best et al. (1998) carried out a retrospective assessment of 262 patients with AAAs with a mean ± SD diameter of 5.5 ± 1.1 cm, who had undergone maximal treadmill exercise stress testing. Of these, one patient (sex/age/demographics not specified) with a large AAA (6.1 cm) was reported to have experienced AAA rupture 12 h after the test, thus a causal link to exercise remains questionable given the temporal delay. Regardless, this yielded a rupture rate of 0.4% that increased to 1% for patients with AAAs > 6.0 cm, and it was concluded that despite theoretical concerns, exercise stress testing of AAA patients is relatively safe, given the low incidence of acute adverse events.

The case report by Robertson et al. (2017) provided the most convincing evidence for a potential link between CPET and AAA rupture in a 77‐year‐old male with an asymptomatic juxtarenal 6.4 cm AAA and a history of emphysema, hypertension and hypercholesterolaemia. Shortly after 11 min of cycle ergometry at a power output of 90 W and HR of 97 beats min^−1^, the test was discontinued owing to breathlessness and fatigue. His immediate post‐test BP was 190/75 mmHg, and 10 min later he complained of bilateral groin/leg pain and decreased sensation in both thighs, with extensive mottling of the lower abdomen and legs, pallor of feet, reduced power in hip and knee flexion and inability to straight leg raise bilaterally. Although haemodynamically stable, CT angiogram (75 min after symptom onset) revealed an intact and stable AAA, leading to diagnosis of gross lower body and spinal cord micro‐embolic phenomena. Follow‐up CT (7 h after symptom onset) confirmed a large retroperitoneal haematoma attributable to AAA rupture, and the patient underwent emergency open surgical repair, surviving to discharge. Although the authors were not in a position to measure the exercise BP response of the patient, they concluded that AAA patients should not avoid CPET owing to risk of rupture, but should be (more) closely monitored for rapid uncontrolled hypertension that should be taken as an indication to terminate the test early (see Section 6 CLINICAL RECOMMENDATIONS: INTEGRATED PHYSIOLOGY‐INFORMED BEST PRACTICE).

## 5 PHYSIOLOGY OF FLOW AND RISK OF RUPTURE: YIN AND YANG

**FIGURE 4:**
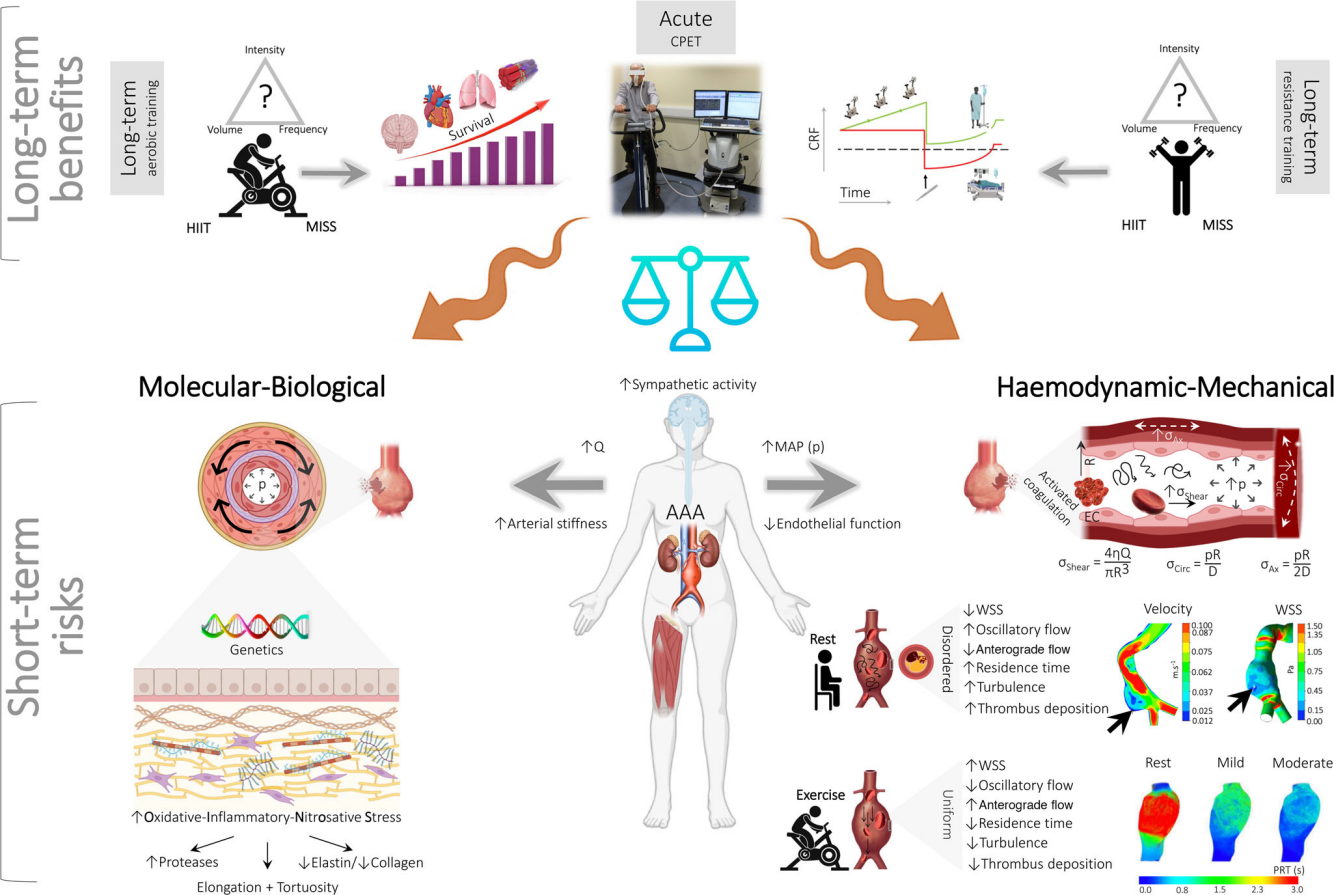
Vascular mechanobiological forces associated with exercise and risk of abdominal aortic aneurysm (AAA) rupture. Long‐term exercise is performed by AAA patients during the course of surgical prehabilitation and typically takes the form of submaximal to (supra)maximal high‐intensity interval training (HIIT). An acute incremental exercise trial to volitional exhaustion is also performed during cardiopulmonary exercise testing (CPET) to assess perioperative risk (Rose et al., 2022). Long‐term benefits: Long‐term moderate‐intensity continuous training (MICT) employing aerobic and/or resistance exercise is generally considered safe and well tolerated, assuming mean arterial pressure (mean aterial pressure (MAP) or pressure (p)) is controlled (see Section 6 Clinical Recommendations), with the capacity to elevate cardiorespiratory fitness (CRF) and improve peri‐operative outcome, including long‐term survival (Haque et al., 2022). Submaximal training in patients with occult small AAAs (3.0–5.5 cm) in the absence of aortic dissection/leak is advocated by respected authorities including, albeit not exclusively limited to, the American Heart Association/American College of Cardiology (Hirsch et al., 2006), Society for Vascular Surgery Practice Guidelines for AAA (Chaikof et al., 2009), Perioperative Exercise Testing and Training Society (Levett et al., 2018) and the American College of Sports Medicine. The American College of Sports Medicine advocates MICT for 20−40 min per session, 3−4 days per week, with an emphasis on duration rather than intensity (Myers, 2009). Short‐term risks: In contrast, concerns have been raised that acute, high(er)‐intensity exercise (notably CPET and potentially HIIT) could potentially tip the balance and precipitate AAA expansion and rupture, which is a catastrophic complication with untreated mortality approaching 100% (Myers et al., 2012; Perissiou et al., 2022). This is primarily attributable to the marked elevation in systemic pressurization combined with complex changes in aortic blood flow topology involving viscous and inertial forces that can adversely impact wall integrity by compounding existing molecular–biological (left) and haemodynamic–mechanical (right) risk factors. Note the complex flow–pressure–strain–shear stress phenotype, including the fundamental equations that govern mechanobiological forces to which endothelial cells in the AAA are exposed (right side). Blood flow (*Q*) in the healthy aorta is primarily unidirectional, laminar anterograde, with high wall shear stress (WSS). In the AAA, flow transitions to a more atherprone phenotype defined by turbulence, increased oscillatory shear stress (OSS) and lower WSS (Tanweer et al., 2014), accelerating oxidative–inflammatory–nitrosative stress (OXINOS)‐mediated structural degeneration of the adventitial wall, diametric expansion and mechanical rupture. Images inset right (modified from Boniforti et al., 2022) include patient‐specific computer tomography images of AAAs, highlighting regions of slow recirculation (longitudinal section) and low WSS (luminal surface) that coincided with the focal region of rupture (black arrows). Flow topology changes substantially with acute exercise owing to altered flow rate waveforms and elevations in Reynolds/Womersley numbers, with WSS increasing >6‐fold in the healthy aorta during moderate‐intensity exercise (50% elevation in heart rate) (Tang et al., 2006). Although calculation of the tensile stresses on the AAA wall and knowledge of corresponding stress failures are technically challenging, emergent evidence, using a combination of patient‐specific imaging and computational fluid dynamic modelling, suggests that acute exercise is atheroprotective owing to higher, more uniform mixing combined with an elevation in WSS and reciprocal reductions in OSS and particle residence time (PRT in mid‐diastole; see inset images modified from Suh et al., 2011) that normalize disordered (basal) flow mechanics (Arzani et al., 2014; Suh et al., 2011; Taylor et al., 1999). Abbreviations: *D*, diameter; *R*, radius; η, viscosity; σ_Ax_, axial stress; σ_Circ_, circumferential stress.

Abdominal aortic aneurysm rupture is a catastrophic consequence of irreversible remodelling of the abdominal aortic wall that occurs instantaneously when the intramural stress borne by the expanding or degenerating aortic wall exceeds its maximum tensile strength, with the latter estimated between 33.6 and 235.1 N cm^−2^ (Raghavan et al., 2006). The predominant source of stress is systemic pressurization that is orders of magnitude greater than the shear stress imparted by blood flow across the aortic wall (Figure 4).

The precise mechanisms that lead to AAA dilatation, adventitial degeneration and eventual rupture are unknown and are likely to involve complex interactions between molecular–biological and haemodynamic–mechanical risk factors (Figure 4). Although current practice for surgical intervention relies on the periodic assessment of geometric changes via ultrasound, CT or magnetic resonance imaging, it fails to account for AAAs that rupture at sizes below operative thresholds, especially in women, and larger AAAs that do not rupture. To improve risk prediction, attention has turned to more advanced patient‐specific *in silico* aortic models that, notwithstanding boundary assumptions and spatial heterogeneity, can provide new insight into pulsatile flow topology and corresponding haemodynamic stresses, such as wall shear stress (WSS), pressure distributions, displacement fields and flow patterns to which the aortic wall is exposed (Mourato et al., 2006).

These approaches have demonstrated that in the healthy aorta of normal aortic diameter (i.e., ∼2 cm), blood flow is typically unidirectional, laminar anterograde and defined by high WSS. In contrast, flow in the dilated rupture‐prone AAA (i.e., approximately, male ≥ 5.5 cm, female ≥ 5.0 cm) is more turbulent and disordered owing to sudden expansion of the flow stream, characterized by low/oscillatory WSS and extended particle clearance times that encourage flow separation, reversal and stagnation on the inner curves and lateral luminal surfaces of aortic bifurcations (Tanweer et al., 2014) (Figure 4). This contributes to atheroprone gene expression and platelet/protease activation, leading to intraluminal thrombus deposition and local hypoxia‐induced OXINOS that can, in turn, lead to arterial pressure waveform amplification and structural degeneration of the adventitial wall, which precede diametric expansion and, ultimately, mechanical rupture (Hossack et al., 2022) (Figure 4).

Acute exercise activates the sympathetic nervous system to elevate cardiac output and BP, increasing aortic blood flow and corresponding WSS, stimulating the original safety concerns over potential AAA rupture. Acute hypertension has also been associated with decreased blood flow to the vasa vasorum, potentially limiting substrate delivery to the media to cause ischaemic weakening of the aortic wall (Heistad et al., 1978). Although basal systemic vascular endothelial dysfunction has been observed in patients with AAA compared with healthy control subjects, confirmed by lower flow‐mediated dilatation (Lee et al., 2017), elevated aortic stiffness (van Disseldorp et al., 2019) and systemic OXINOS (Bailey et al., 2006, 2022), there are, to the best of our knowledge, no published studies that have determined whether sympathetic outflow and corresponding BP responses, either at rest or during exercise, are augmented as a consequence. On the contrary, the BP response to exercise appears to be normal [i.e., equivalent to age‐matched controls (Bailey et al., 2018) or when normalized relative to energy expenditure (Myers et al., 2011)].

This is surprising, given that human thoracic aortic dissection and peripheral arterial disease, which are typically associated with AAA disease (Takagi et al., 2016), are characterized by elevations in basal and exercise‐induced systemic and regional aortic sympathetic activity (Qin et al., 2022; Zhipeng et al., 2014). Indeed, an exaggerated exercise pressor reflex, a peripheral neural reflex originating in skeletal muscle that encompasses the metaboreflex and the mechanoreflex (Smith et al., 2006), contributes to the more marked elevation in (exercise‐induced) muscle sympathetic nerve activity and BP consistently observed in patients with peripheral arterial disease (Baccelli et al., 1999; Cui et al., 2021). The increases in muscle sympathetic nerve activity in peripheral arterial disease occurred early and were much greater than those at the same exercise time/workload in matched healthy control subjects. Future studies are encouraged to determine the extent to which the exercise pressor reflex and related components underlying the integrated regulation of sympathetic outflow differ in AAA patients, notably the central command and the arterial/cardiopulmonary baroreflexes (Katayama & Saito, 2019).

However, these changes are countered by an exercise intensity‐dependent endothelial release of shear‐induced atheroprotective molecules, such as NO and prostacyclin, that collectively reverse the (basal) atheroprone flow phenotype (Taylor et al., 1999). In support, computational fluid mechanics modelling approaches have consistently demonstrated more undirectional anterograde flow, with almost complete suppression of retrograde flow, combined with reduced stagnation and oscillatory WSS, during mild‐ to moderate‐intensity lower‐limb exercise (Arzani et al., 2014; Suh et al., 2011; Tang et al., 2006; Taylor et al., 1999; Zhan et al., 2022) (Figure 4). The mechanovascular transition from an atheroprone to a more atheroprotective flow phenotype appears to be related to the penetrating blood jet in systole (Arzani et al., 2014). The corresponding mechanobiological forces imposed by blood flow are sensed and subsequently transduced by endothelial nuclei stimulating focal suppression of OXINOS and corresponding intramural thrombus deposition/structural wall degeneration (Salvador & Iruela‐Arispe, 2022). This provides a mechanistic rationale to explain the long‐term vascular benefits, including attenuation in AAA growth, associated with MICT (Nakayama et al., 2018) that can be potentiated by HIIT.

## 6 CLINICAL RECOMMENDATIONS: INTEGRATED PHYSIOLOGY‐INFORMED BEST PRACTICE

Previous publications, albeit with limited guidance, have advocated a conservative approach when conducting preoperative CPET in AAA patients. For example, concern is predicated by the potential for an increased risk of a cardiac event and/or BP‐induced rupture because the majority of AAA patients (60%–70%) exhibit a higher incidence of comorbid cardiac disease in comparison to other age‐matched surgical populations (Tew et al., 2014). However, as outlined, exercise performed under clinical supervision is generally considered safe in the absence of hypertension, although there is currently no consensus regarding the safe upper limit beyond which BP should not be allowed to continue to rise and CPET should be terminated.

The Association for Respiratory Technology and Physiology Statement on Cardiopulmonary Exercise Testing 2021 recommends a conservative approach for AAA patients undergoing CPET, and that exercise should be discontinued if BP exceeds 200 mmHg systolic or 110 mmHg diastolic and that consideration should be given to submaximal testing for determination of GET (Pritchard et al., 2021). This might prove attractive for patients with musculoskeletal disorders who are physically unable to attain cardiovascular limits (e.g., HR within age‐predicted maxima) owing to muscle fatigue or pain.

However, emerging evidence suggests that a maximal effort is preferable, given that it provides superior risk prediction (2‐year mortality) in AAA patients, with the area under receiver operating characteristic curves shown to be higher for ${\dot{V}}_{O_{2}\mathrm{peak}}$ compared with GET (0.71 vs. 0.65 with cut‐points of 13.1 mL kg^−1^ min^−1^ and 34 L min^−1^ L min^− 1^, respectively; Rose, Davies, Appadurai et al., 2018), probably attributable to the comparatively lower biological variability associated with ${\dot{V}}_{O_{2}\mathrm{peak}}$ (Rose, Davies, Davison et al., 2018). These (BP) upper limits are considerably lower than those advocated by other respected bodies during exercise stress testing of patients with established cardiovascular or pulmonary disease (250 mmHg systolic or 120 mmHg diastolic; American Thoracic Society, 2003; Radtke et al., 2019) but greater than those documented in two of the aforementioned cases of (potentially exercise‐related) AAA rupture (Puls & Thadani, 1986; Robertson et al., 2017). As discussed, other investigators have advocated lower boundary thresholds, not during CPET itself, but over the course of long‐term training that also extends to HIIT (Tew et al., 2014). However, these safety recommendations might be considered overly conservative, with the upper (systolic) BP limit probably preventing AAA patients from exercising to volitional exhaustion and achieving a peak/maximal effort (i.e., when CPET is terminated by the physiologist before the patient reaches their symptom limitation) (Sabbahi et al., 2018).

Systolic BP is expected to rise with exercise and is a normal physiological response. The hypertensive response to exercise has been defined as systolic BP ≥210 mmHg in men or ≥190 mmHg in women or diastolic BP ≥110 mmHg in men or women (Schultz & Sharman, 2014) defined from normative data (Daida et al., 1996). More recent exercise BP data from the FRIEND national fitness registry suggests that these peak cut‐off values might be too conservative and should be revised using age‐specific norms (Sabbahi et al., 2018). However, to conduct CPET safely in patients with AAA, it would seem prudent to avoid an excessive hypertensive response to exercise.

It is equally unclear to what extent resting and recovery haemodynamic responses to CPET, measures that are routinely assessed yet rarely used clinically during CPET, can further inform risk prediction/test termination in AAA patients, given a general lack of research. There is physiological rationale, given that elevated resting HR, chronotropic incompetence and attenuated HR recovery collectively reflect autonomic nervous system dysfunction. In support, elevated preoperative resting HR (>96 beats min^−1^) has been associated with postoperative myocardial injury and mortality within 30 days after non‐cardiac surgery (Abbott et al., 2016). An exaggerated HR response during unloaded cycling (>12 beats min^−1^) has also been associated with an increased risk of myocardial ischaemia, reduced cardiac performance (lower O_2_ pulse as a surrogate for left ventricular volume) and prolonged hospital stay (Whittle et al., 2015). All‐cause morbidity within 5 days of non‐cardiac surgery was also elevated in patients exhibiting impaired cardiac vagal function (HR recovery of <12 beats min^−1^ after preoperative CPET; Ackland et al., 2019). Equally, chronotropic incompetence, reflecting the inability to increase HR and match cardiac output to metabolic demands, is a predictor of major adverse cardiovascular events in patients with coronary artery disease (Lauer et al., 1999), although its predictive application in the surgical setting (AAA not specified), including prevalence and cut‐offs, remains equivocal (Otto et al., 2020). However, given the high prevalence of ischaemic disease in AAA (Greenhalgh et al., 2004), a steep HR–${\dot{V}}_{O_{2}}$ slope and/or sudden increase in slope (consistent with static or falling stroke volume) might prove an independent risk factor potentially relevant to CPET risk mitigation and early termination.

Equally, there is a general lack of consensus relating to safe upper limits of AAA diameter pertaining to CPET, although >8.0 cm has been recommended as a relative contraindication (Levett et al., 2018). However, it is unclear what evidence, if any, has informed this criterion. This cut‐off might well have originated from guidelines for the treatment of AAAs of the Joint Council of the American Association for Vascular Surgery and Society for Vascular Surgery in 2003, which estimated that the highest annual rupture risk was in aneurysms >8.0 cm (∼30%–50% year^−1^) (Brewster et al., 2003). Fortunately, aneurysm diameters >8.0 cm appear to be rare, with a reported prevalence of 0.03% (Lederle et al., 2000). However, in this subset of patients it can be argued that a number of them will present symptomatically and therefore require urgent repair, not allowing for CPET, although they would benefit from regular examinations, including baseline pulmonary and cardiac function. These arguments further highlight the need for definitive expert guidelines.

**FIGURE 5:**
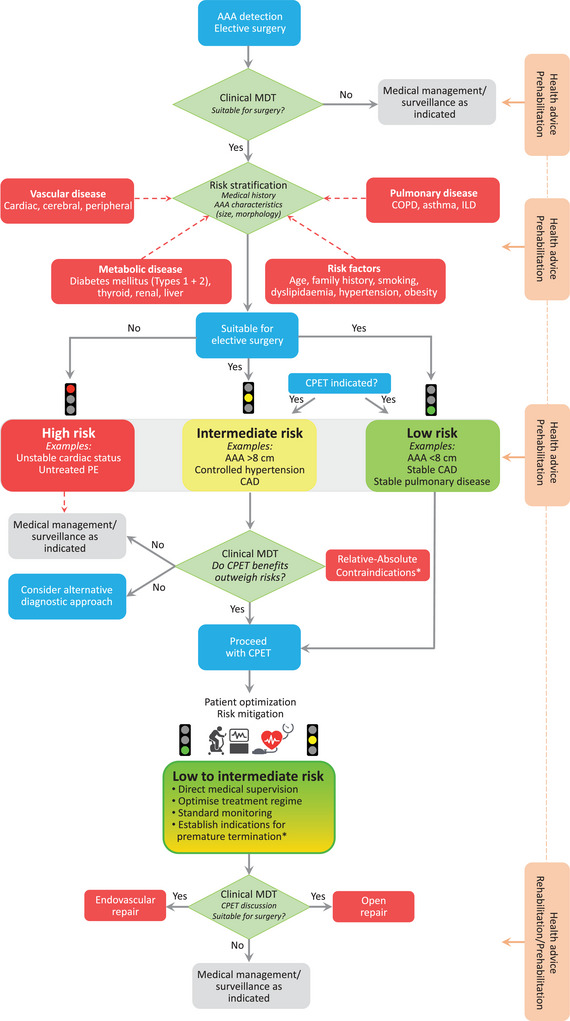
Modified algorithm based on previously published guidelines (ACSM, 2010; American Thoracic Society, 2003; Laveneziana et al., 2021; Levett et al., 2018; Pelliccia et al., 2021; Pritchard et al., 2021; Radtke et al., 2019), highlighting a systematic approach to clinical stratification, precautions, optimization and corresponding risk mitigation during cardiopulmonary exercise testing (CPET) in patients with an abdominal aortic aneurysm (AAA). Note that CPET data should be interpreted by a specialist and functionally integrated with other clinical findings, including surgical/anaesthetic factors, AAA diameter/morphology and resting/exercise blood pressure responses, with the last of these monitored continuously (as highlighted). *Please refer to Table 1. Abbreviations: CAD, coronary artery disease; COPD, chronic obstructive pulmonary disease; EVAR, endovascular repair; ILD, interstitial lung disease; MDT, multidisciplinary team; PE, pulmonary embolus. Turndown refers to a patient deemed unsuitable for surgical repair of AAA.

Unless immediate surgery is planned, a cardiac magnetic resonance angiogram is preferred for AAA patients. A modified algorithm based on previously published guidelines (ACSM, 2010; American Thoracic Society, 2003; Laveneziana et al., 2021; Levett et al., 2018; Pelliccia et al., 2021; Pritchard et al., 2021; Radtke et al., 2019), highlighting a systematic approach to (AAA) clinical stratification, precautions, optimization and corresponding risk mitigation during CPET, is illustrated in Figure 5. This is complemented by a summary of the absolute and relative contraindications to CPET based on modification of (prior) published recommendations, taking into account the specifics of the AAA patient population (Table 1). These recommendations are especially relevant for patients with chronic obstructive pulmonary disease, given their elevated (basal) AAA rupture risk (hazard ratio, 1.7; 95% confidence interval, 1.5−1.9), which persists even after adjustment for common risk factors, including documented tobacco use (Mahta et al., 2019). This might simply reflect ‘shared’ pathogenic pathways characterized by an imbalance in protease/antiprotease activity that predisposes to focal OXINOS and subsequent connective tissue degradation in both alveolar walls (Lomas, 2016) and aneurysmal walls (Bailey et al., 2006, 2022). Furthermore, the development of exercise‐induced dynamic hyperinflation and sinusoidal swings in intrathoracic pressure to overcome the increased elastic and resistive loads (Boerrigter et al., 2014) could further compound the risk of CPET‐induced AAA rupture, highlighting the need for extra vigilance in patients with chronic obstructive pulmonary disease.

**TABLE 1:** Contraindications and premature termination during cardiopulmonary exercise testing in patients with an abdominal aortic aneurysm.

Contraindications	
**Absolute**	
Recent significant change in resting ECG reflecting significant ischaemia, recent myocardial infarction (within 2 days) or other acute cardiac event Unstable angina Uncontrolled cardiac arrhythmias causing symptoms or haemodynamic compromise Syncope Active endocarditis, myocarditis or pericarditis Active pulmonary embolus or pulmonary infarction Symptomatic severe aortic stenosis Uncontrolled symptomatic heart failure Suspected dissecting or leaking aortic aneurysm Uncontrolled asthma Arterial desaturation at rest on normoxic room air, $S_{pO_{2}}$ < 85% Acute systemic infection, accompanied by fever, body aches or swollen lymph nodes Severe musculoskeletal compromise	
**Relative**	
Left main stem coronary stenosis Moderate stenotic valvular heart disease Electrolyte abnormalities (e.g., hypokalaemia, hypomagnesaemia) Tachyarrhythmias or bradyarrhythmias Hypertrophic cardiomyopathy and other forms of outflow tract obstruction Neuromuscular, musculoskeletal or rheumatoid disorders exacerbated by exercise High‐degree atrioventricular block Ventricular aneurysm Uncontrolled metabolic disease (e.g., diabetes, thyrotoxicosis or myxoedema) Severe untreated arterial hypertension at rest (systolic BP >200 mmHg, diastolic BP >120 mmHg) Significant pulmonary hypertension Thrombosis of the lower extremity until treated for a minimum of 2 weeks Advanced or complicated pregnancy Chronic infectious disease (e.g., mononucleosis, hepatitis, AIDS) Mental or physical impairment leading to inability to exercise adequately	
**Early termination**	
Angina >2 mm ST depression if symptomatic or 4 mm if asymptomatic or >1 mm ST elevation Significant arrhythmias causing symptoms or haemodynamic compromise Fall in systolic blood pressure >20 mmHg from the highest value during the test Hypertension (systolic BP ≥210 mmHg in males or ≥190 mmHg in females or diastolic BP ≥110 mmHg in males or females) Severe arterial desaturation: $S_{pO_{2}}$ < 80% (lower may be accepted in patients with known underlying lung disease) Loss of coordination Mental confusion Dizziness or faintness	

Abbreviations: AIDS, acquired immune deficiency syndrome; BP, blood pressure; ECG, electrocardiogram; $S_{pO_{2}}$, peripheral oxygen saturation.

## 7 CONCLUSIONS

Preoperative CPET is an excellent tool for objective evaluation of patient CRF and individualized risk stratification. Recommendations for exercise testing patients with AAA are lacking. Bringing together a multidisciplinary group of physiologists, exercise scientists, anaesthetists, radiologists and surgeons, this review challenges the enduring ‘myth’ that AAA patients should be fearful of and avoid rigorous exercise. On the contrary, by appraising fundamental vascular mechanobiological forces associated with exercise together with ‘methodological’ recommendations for risk mitigation specific to this patient population, we highlight that the benefits conferred by CPET and exercise training across the continuum of intensity far outweigh the short‐term risks posed by potential AAA rupture.

## AUTHOR CONTRIBUTIONS

Damian M. Bailey conceived the idea and wrote the first draft of the manuscript and corresponding revisions. Richard G. Davies, George A. Rose, Chistopher P. Twine, Matti Jubouri, Joseph S. Coselli, Mohamad Bashir and Ian M. Williams revised the manuscript. Damian M. Bailey approved the final version submitted for publication and agrees to be accountable for all aspects of the work in ensuring that questions related to the accuracy or integrity of any part of the work are appropriately investigated and resolved. All persons designated as authors qualify for authorship, and all those who qualify for authorship are listed.

## CONFLICT OF INTEREST

Damian M. Bailey is Editor‐in‐Chief of *Experimental Physiology*, Chair of the Life Sciences Working Group, a member of the Human Spaceflight and Exploration Science Advisory Committee to the European Space Agency, a member of the Space Exploration Advisory Committee to the UK Space Agency, and a member of the National Cardiovascular Network for Wales and South East Wales Vascular Network. Damian M. Bailey is also affiliated to the companies FloTBI Inc. and Bexorg Inc., focused on the technological development of novel biomarkers of brain injury in humans. Mohamad Bashir is a Consultant for Terumo & JOTEC/CryoLife. Ian M. Williams is a member of the South East Wales Vascular Network. Chistopher P. Twine is a member of the Bristol Bath and Weston Vascular Network. Joseph S. Coselli is affiliated with the companies Terumo Aortic, Medtronic, W.L. Gore & Associates, Abbott Laboratories, CytoSorbents, Edwards Lifesciences and Artivion.
